# Roles of phosphatidyl inositol 3 kinase gamma (PI3Kγ) in respiratory diseases

**DOI:** 10.15698/cst2021.04.246

**Published:** 2021-03-08

**Authors:** Valentina Sala, Angela Della Sala, Alessandra Ghigo, Emilio Hirsch

**Affiliations:** 1 Department of Molecular Biotechnology and Health Sciences, University of Torino, Via Nizza 52, 10126, Torino, Italy.; 2 Kither Biotech S.r.l. Via Nizza 52, 10126, Torino, Italy.; # Equal contribution to senior authorship

**Keywords:** PI3K signaling, chronic respiratory disease, restrictive airway disease, obstructive airway disease, inflammation

## Abstract

Phosphatidyl inositol 3 kinase gamma (PI3Kγ) is expressed in all the cell types that are involved in airway inflammation and disease, including not only leukocytes, but also structural cells, where it is expressed at very low levels under physiological conditions, while is significantly upregulated after stress. In the airways, PI3Kγ behaves as a trigger or a controller, depending on the pathological context. In this review, the contribution of PI3Kγ in a plethora of respiratory diseases, spanning from acute lung injury, pulmonary fibrosis, asthma, cystic fibrosis and response to both bacterial and viral pathogens, will be commented.

## INTRODUCTION

Phosphatidyl inositol 3 kinases (PI3Ks) are a family of lipid kinases that play key roles in a plethora of processes, including cell growth, proliferation and differentiation, tissue morphogenesis, metabolism, and immune function. The PI3K family is divided into three classes with distinct functions, among which the best characterized is class I, which phosphorylates phosphatidylinositol 4,5 bisphosphate in the third position to generate the second messenger phosphatidylinositide 3,4,5 trisphosphate. Class I PI3K subfamily is further divided into two classes: class IA, which is composed of α, β and δ isoforms, and class IB, whose only member is PI3Kγ [1]. Class I PI3K isoforms display different expression patterns: while PI3Kα and PI3Kβ are ubiquitously expressed, PI3Kδ and PI3Kγ have a more restricted expression pattern. Accordingly, deficiency in PI3Kα or PI3Kβ is embryonic lethal in murine models, whereas PI3Kγ or PI3Kδ knockout (KO) mice are viable and fertile [2].

In particular, PI3Kγ is expressed, at very low levels under physiological conditions, in cell types including cardiomyocytes [3–9], vascular smooth muscle cells [10], and the microglia [11], where it is significantly upregulated after stress.

On the contrary, PI3Kγ is constitutively enriched in leukocytes (neutrophils, eosinophils, macrophages, T cells and mast cells) [12]. Consistently, PI3Kγ KO mice exposed to natural pathogens/microbiota display altered immune traits that closely mirror the human Inactivated PI3Kγ Syndrome (IPGS) [13]. Intriguingly, clinical signs related to loss of PIK3γ include autoimmune cytopenia and infections, as well as pathological infiltration of T cells in barrier organs, including the lungs, that are hyper-responsive to microbial products [13].

Of utmost relevance for respiratory homeostasis and disease, PI3Kγ is also expressed in all the other cell types that are involved in airway disease, like endothelial cells [14], fibroblasts [15], and epithelial cells [16, 17].

Besides such diverse expression patterns, class I PI3Ks own non-redundant roles in the response to a variety of stimuli. Class IA PI3Ks, exception done for PI3Kβ that can be also activated by G-protein Coupled Receptors (GPCRs) [18], are recruited to receptor tyrosine kinases through the SH2 domains of p85-like regulatory subunits. Class IB PI3Kγ is composed of the p110γ catalytic subunit, and of the p101 and p84/p87 subunits. These two adapter companions have important non-redundant roles in coupling PI3Kγ to upstream Ras/GPCRs signaling pathways [19]. While p84 is a component of a constitutively-expressed PI3K complex, p101 is part of an inducible PI3K complex [20]. Moreover, the p110γ/p84 heterodimer is less sensitive to the activation promoted by Gβγ subunits and depends on Ras partnership, while activation of the p110γ/p101 variant by Gβγ subunits is more favorable and Ras-independent [21]. Importantly, p110γ acts as an A-kinase anchoring protein (AKAP), being engaged in a functional and physical interaction with PKA that does not involve its kinase activity [7, 8]. Thus, PI3Kγ is not only a kinase but also a scaffold protein for PKA in a complex containing type 3 and 4 phosphodiesterases (PDEs). This complex acts in a negative feedback loop, suppressing cyclic adenosine monophosphate (cAMP) levels in the vicinity of the β2-adrenergic receptor, through PKA-mediated activation of PDEs [7, 8].

Therefore, acting at the crossroads of multiple pathways [1, 22], PI3Kγ is a hub of intracellular signaling. As an example, PI3Kγ is activated downstream of GPCRs by both metabolic signals acting on β-adrenergic receptors, and immune signals like chemokines and complement fragments. Moreover, PI3Kγ can be activated by pathogen- and damage-associated molecular patterns downstream of Toll-like receptors (TLRs) in myeloid cells [23–25] and cardiomyocytes [5], functioning as a master regulator at the interface between metabolic and immune homeostasis. The relevance of the PI3Kγ hub as a regulator and amplifier for diverse and converging signaling pathways is evident in mast cells, where the FcεRI receptor mediates PI3Kγ activation. Yet, the FcεRI receptor has no direct link to GPCRs, but degranulation relies on PI3Kγ [26]. Intriguingly, the combinatorial regulation of PI3Kγ heterodimer variants can lead to a remarkable level of signaling specificity, which depends on both the tissue and the physio-pathological context [27].

**TABLE 1.** Differential responses of PI3Kγ KO mouse models of respiratory diseases.

**Pathology**	**PI3Kγ KO phenotype**	**References**
Airway inflammation	Within all studies, PI3Kγ-deficient mice are healthy and viable with reduced allergic AHR, inflammation, and remodelling. In the absence of PI3Kγ, the chemokine-induced model of airway inflammation displays impaired neutrophils, eosinophils and macrophages chemotaxis, reduced peribronchial fibrosis and TGF-β1+ cells and lower Smad 2/3 signaling.	[57, 91, 116]
Lung injury, Fibrosis	PI3Kγ deficiency confers protection against bleomycin-induced pulmonary injury. PI3Kγ KO mice display reduced weight loss, decreased lethality, reduced deposition of lung collagen and lower expression of profibrogenic and proangiogenic genes.	[51]
Lung injury, Inflammation	PI3Kγ KO mice display reduced accumulation of neutrophils in an LPS-induced acute lung injury model, and perturbation in E-selectin-mediated adhesion, in response to TNF-α.	[117]
Lung injury, Endotoxemia	Endotoxemia-induced lung edema, neutrophil accumulation, nuclear translocation of NF-κB and production of proinflammatory cytokines (IL-1β and TNF-α) in lung neutrophils are reduced in transgenic mice lacking the catalytic subunit of PI3Kγ.	[118]
Lung injury, ARDS	In acute lung injury and adult respiratory distress syndrome (ARDS) models, PI3Kγ KO mice display reduced histological evidence of lung injury after high volume ventilation and reduced PKB phosphorylation compared to wild-type, independently from inhibitory effects on cytokine release.	[16, 119]
Lung vascular injury, Inflammation	In a model where lung vascular injury was induced by bacteraemia (i.e. by intraperitoneal *Escherichia coli* injection), PI3Kγ KO mice present higher levels of leucocyte accumulation in the lung, and greater microvascular permeability, resulting in lung edema. These results point to PI3Kγ as a negative regulator of lung vascular injury in gram-negative sepsis.	[120]

**Figure 1:**
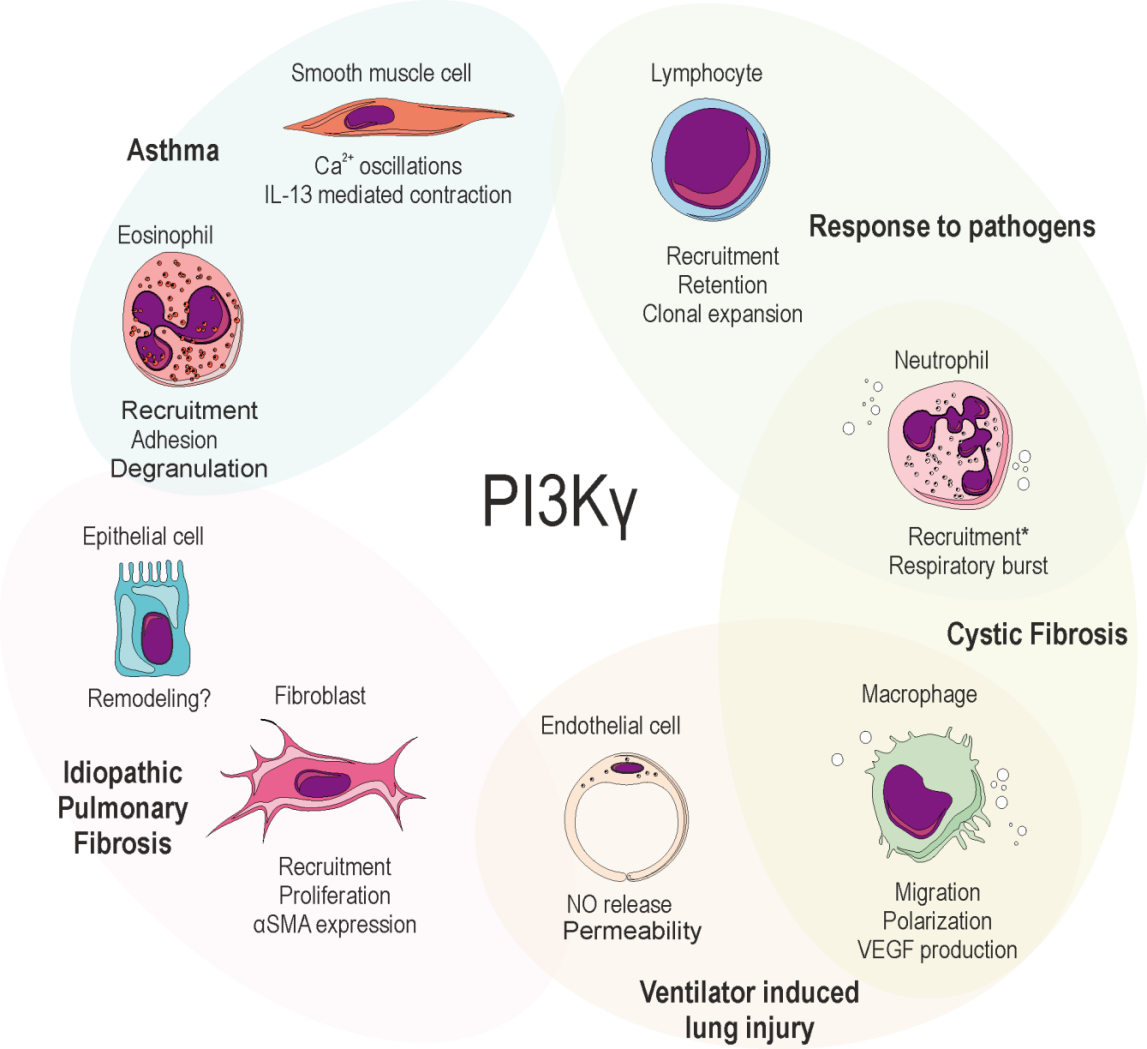
FIGURE 1: Schematic representation of the biological processes induced or mediated by PI3Kγ in the cell types that are relevant for respiratory disease. PI3Kγ has been involved in almost all target cells shown in the figure using either isoform-selective PI3K inhibitors or genetic engineering. *Neutrophilic recruitment may be enhanced by either activation or inhibition of PI3Kγ, depending on the context. NO: Nitric Oxide; αSMA: α-smooth muscle actin; VEGF: Vascular endothelial growth factor; IL-13: Interleukin-13.

Moreover, studies demonstrating the effects of knocking out PI3Kγ in murine disease models (**Table 1**) led to great interest in the immunological functions and in the potential of PI3Kγ as a therapeutic target in inflammatory-driven diseases [15], including those affecting the airways. Within this review, we intend to highlight the relevance of PI3Kγ as a trigger or target in a plethora of respiratory diseases, spanning from acute lung injury, pulmonary fibrosis, asthma, cystic fibrosis and response to both bacterial and viral pathogens (**Figure 1**).

## PI3Kγ ROLE IN RESPIRATORY DISEASES

### Ventilator-induced damage

Mechanical ventilation is a life-saving therapy but can contribute to the progression or even initiate lung injury *per se*. Ventilation-induced lung injury (VILI) clinically displays signs of alveolar edema, including increased vascular permeability and accumulation of fluids in the alveoli [28].

In VILI, the mechanical stress induced by ventilation activates the inflammasome in macrophages and endothelial cells, leading to enhanced nitric oxide (NO), oxygen radicals, and peroxynitrite production, which contributes to the increase of alveolar and vascular permeability [28] and impairs alveolar fluid clearance [29]. In line with these findings, the inhibition of PI3Kγ kinase activity specifically in resident lung cells attenuates VILI through the reduction of NO release [30].

Besides NO, the intracellular level of cAMP is critical for the modulation of endothelial permeability [31]. Since PI3Kγ can act as a scaffold, independently of its kinase activity, to modulate cAMP levels [7, 8], its role in the formation of edema during VILI has been investigated. Accordingly, PI3Kγ knockout lungs are protected from VILI [16]. Moreover, pharmacological combined regimens aimed at blocking PI3Kγ kinase activity while increasing cAMP levels attenuate VILI in PI3Kγ wild-type lungs deprived of circulating leukocytes [30], pointing to the central role of PI3Kγ in lung cell types other than the immune cells.

Nevertheless, PI3Kγ is largely expressed in leukocytes, whose contribution to Vascular endothelial growth factor (VEGF) production, inflammation and injury in VILI is recognized [32]. A major player in the control of local and systemic immune response is TLR4 [33, 34]. Notably, it has been shown that TLR4 is overexpressed [35], and has a key role in experimental models of VILI [36–39]. In macrophages, upon TLR4 receptor activation, PI3Kγ is recruited by Rab8, and is required to activate the Akt/mTOR pathway to bias the cytokine response towards an anti-inflammatory scenario [24]. Therefore, these findings suggest that the contribution of the TLR4/PI3Kγ axis to VILI pathogenesis deserves further investigation.

PI3Kγ emerged therefore as a possible therapeutic target in the treatment and/or prevention of VILI and edema. However, strategies aimed at blocking PI3Kγ kinase dependent and independent activities only in resident inflammatory and parenchymal lung cells, such as airway epithelial cells, should be addressed. This could enable to mitigate potential systemic side effects on the innate immune system on one side, and on different cAMP-responsive cells, like cardiomyocytes, on the other.

### Idiopathic pulmonary fibrosis (IPF)

Idiopathic pulmonary fibrosis (IPF) is a chronic, progressive, fibrotic interstitial lung disease of unknown etiology, which occurs primarily in older adults (median age at diagnosis is about 65 years) [40]. Although it is classified as a rare disease (occurring in less than 5/10,000 persons per year), IPF is the most common type of idiopathic interstitial pneumonia, occurring with a frequency comparable to that of stomach, brain and testis cancers [41]. Moreover, the global burden of IPF is extremely high, due to the poor prognosis, with a median survival time of two to four years from diagnosis [42].

Historically, IPF was considered a chronic and progressive inflammatory disorder, which gradually leads to established fibrosis. However, the failure of anti-inflammatory therapies [43] caused a profound revision of this concept [44]. IPF is now thought to result from the concomitance of repetitive local micro-injuries to the ageing alveolar epithelium, genetic factors [45], and environmental risk factors (such as cigarette smoke, drugs, lung microbiome, infections or environmental pollutants) [42, 46]. In turn, intracellular signaling initiated by micro-injuries gives rise to an aberrant communication between epithelial cells and fibroblasts, leading to increased extracellular matrix accumulation, and, ultimately, to lung interstitial remodeling and loss of function.

Within this context, PI3K signaling emerged as a crucial pathway in models of pulmonary fibrosis [47, 48]. In particular, class I PI3Ks play key roles in the homeostasis of all the cell types that are involved in the pathogenesis of IPF. Consistently, an inhaled pan-Class I PI3K inhibitor has been demonstrated to have a protective effect against the rapid, progressive pulmonary fibrosis induced by instillation of bleomycin *in vivo* [48], by reducing the expression of pro-fibrotic genes, including transforming growth factor-β (TGF-β) and connective tissue growth factor (CTGF) [49]. Among class I isoforms, PI3Kγ is overexpressed in myofibroblasts and bronchiolar basal cells in the lungs of IPF patients, and, *ex vivo*, in human IPF primary fibroblasts [50]. Both genetic and pharmacological inhibition of PI3Kγ are able to inhibit proliferation as well as α-smooth muscle actin (αSMA) expression in IPF fibroblasts *in vitro* [50]. Accordingly, mice lacking PI3Kγ are protected from the accumulation of matrix and leukocytes in the lungs after bleomycin injury [51], pointing to PI3Kγ as a promising therapeutic target for IPF.

Recently, the need of pathway-specific biomarkers and genetic phenotyping has emerged in order to identify patient subtypes for new combinatorial trials [52]. In fact, due to its intrinsic complexity, the natural history of IPF is highly variable and the course of the disease in an individual patient is somewhat unpredictable, as some patients experience a rapid lung decline, while others progress much more slowly. Of note, a rapidly progressive disease has been associated with the upregulation of several genes, including TLR9 [53], downstream of which PI3Kγ is activated, at least in cardiomyocytes [5]. Moreover, in the past two decades, metabolic dysregulation, impaired mitochondrial autophagy, and mitochondrial dysfunction have been observed in cells of IPF lungs [54].

Overall, these results suggest the intriguing hypothesis that the activation level of PI3Kγ might act as a master controller in the different processes that converge on IPF pathogenesis and influence the fate of the lung environment. Whether PI3Kγ will be a suitable biomarker or therapeutic target in IPF patients, however, still has to be investigated.

### Asthma

The role of PI3K family members in asthma is well documented and pan-class I PI3K topical inhibition is effective against acute and, more importantly, glucocorticoid resistant asthma [49]. Focusing on the specific contribution of PI3Kγ to asthma pathogenesis, KO of PI3Kγ or treatment with an aerosolized dual inhibitor of PI3Kγ and δ (TG100-115), is able to reduce eosinophilic airway hyper-responsiveness (AHR) and inflammation in experimental models [54–57].

Moreover, the PI3Kγ-specific inhibitor AS605240 dampens eosinophilic inflammation induced by the CC chemokine eotaxin (CCL11), by suppressing signaling pathways downstream of CC chemokine receptor 3 (CCR3) [58]. In detail, AS605240 inhibits eotaxin-induced chemotaxis, adhesion to Intercellular Adhesion Molecule 1 (ICAM-1), and degranulation of human peripheral blood eosinophils by inhibiting ERK1/2 phosphorylation, without down-regulation of surface CCR3 expression [58].

Mechanistically, the pathological role of PI3Kγ in asthma implicates the release of inflammatory cell mediators, including macrophage migration inhibitory factor (MIF) and the T-helper type II cytokine Interleukin-13 (IL-13).

MIF participates as a proinflammatory cytokine in both innate and adaptive immune responses, contributing to the pathogenesis of inflammatory, metabolic, autoimmune, and allergic diseases. Of note, MIF plays a pivotal role in activating the expression of PI3Kγ regulatory (p101) and catalytic subunits (p110) [59]. In turn, increased PI3Kγ activity is responsible for IL-13-mediated contraction of airway smooth muscle (ASM) cells, the underlying mechanism of AHR induced by allergen sensitization or cytokines in asthma [59–63]. IL-13 receptor and PI3Kγ are both expressed in ASM cells, in which they control contractility by regulating Ca^2+^ oscillations [64]. Notably, IL-13, which is increased in the airways of asthmatic patients and correlates with AHR [65], is sufficient [66] and required [67] for the development of allergen-induced AHR. In a translational perspective, targeting PI3Kγ, either pharmacologically or by RNA interference, suppresses IL-13-dependent contractility of ASM cells, and, more importantly, intranasal administration of a PI3Kγ inhibitor attenuates IL-13-induced AHR in mice [64]. Therefore, dampening IL-13 levels by targeting the upstream PI3Kγ signaling might be a feasible and efficient strategy to reduce Ca^2+^ oscillations and contraction in ASMs.

Overall, these data underline the promising therapeutic potential of PI3Kγ inhibition in asthma [68].

### Cystic Fibrosis

Cystic fibrosis (CF) is the most common genetic disease in the Caucasian population, affecting ~1 in 3,500 persons. The basic defect of CF results from mutations in a single gene encoding for the CF transmembrane conductance regulator (*CFTR*), a 1,480 residues transmembrane glycoprotein that regulates cAMP-mediated chloride (Cl^-^) conductance at the apical surface of secretory epithelia. Impaired secretion of Cl^-^ and bicarbonate triggers dehydration of the airway surface liquid, resulting in increased mucus viscosity and impaired mucociliary clearance. The accumulated mucus ultimately favors colonization by pathogens and resistance to treatments [69]. In turn, airway mucus obstruction and recurrent/persistent bacterial infections trigger a chronic neutrophilic inflammation, which are responsible for the release of neutrophilic elastases and for the ensuing, progressive lung damage and decline of function in CF patients [70].

In this context, the inflammatory response in CF lungs is non-resolving and self-perpetuating. In fact, the vicious cycle of neutrophilic burden and release of noxious mediators, further fuels inflammation and infection, and further contributes to disease progression towards irreversible lung damage. Notably, albeit chronic bacterial infections play a prominent role in the progression of CF lung disease, inflammation was observed in the lungs of asymptomatic CF infants without any apparent established bacterial infection [71], suggesting that sterile inflammation can precede, and possibly promote, infection in early-stage CF lung disease, by favoring the expansion of more pathogenic strains among the lung microbiota. Consistently, recent studies suggest that, upon migration to CF airways, neutrophils undergo a phenotypic reprogramming, leading to dysregulated lifespan, metabolism and effector function, ultimately contributing, together with the epithelium and resident microbiota, to the evolution of a pathological microenvironment [72].

Therefore, anti-inflammatory therapy, eventually combined with antibiotics, is crucial to prevent lung damage. However, currently used therapeutic strategies show limited clinical benefit. With the aim of filling this gap, the possibility to interfere with leukocyte trafficking into CF airways has been explored. PI3Kγ has a key role in this process, triggering signaling pathways evoked by binding of chemotactic factors to GPCRs. Among these, IL-8 represents the principal neutrophil chemoattractant and its elevated concentration characterizes CF lung inflammation. In the CF context, the biological efficacy of both genetic deletion and pharmacological inhibition of PI3Kγ in reducing chronic neutrophilic inflammation in the lungs has been demonstrated in *β*-ENaC overexpressing CF-like mice [73].

While most research on CF inflammation has focused on epithelial cells and neutrophils, macrophages play an important role in the initiation and resolution of pulmonary inflammation. Functional abnormalities have been observed in CF macrophages from experimental models, including newborn CF pigs, and from CF patients, and found to display a constitutive proinflammatory status and hyper-responsiveness to microbial stimuli, supporting the presence of a primary defect in CF macrophages, which seems to be correlated to CFTR channel function [74, 75].

Of note, mucus stasis *per se* might be responsible for the pro-inflammatory polarization of airway macrophages [76], albeit data from CF patients point to a CFTR-dependent defect in the resolution phase of inflammation, due to the inability of CF macrophages to re-polarize to the M2 immunosuppressive phenotype [77]. Notably, blockade of PI3Kγ activity promotes M1 macrophage polarization in implanted tumors, and inflammation, albeit M2 polarization has been observed in obese mice lacking PI3Kγ [78], suggesting that the cellular context and activation level of PI3Kγ might be crucial to determine the fate of macrophages.

Overall, these data would support the relevance of the immune response in CF disease, but whether abnormalities in immune cells, including changes to macrophage polarization, could be corrected using CFTR-directed therapies remains an open question. Whereas blockade of PI3Kγ activity by small-molecule inhibitors may represent a valid approach to down-modulate neutrophil recruitment and burst in inflamed tissues, the resulting increased susceptibility to infection might be a potential side effect. Therefore, focused therapeutic windows should be defined for the use of these molecules in CF patients.

## PI3Kγ IN INFECTIVE DISEASES

### Bacterial infections

*Streptococcus pneumoniae* is the most prevalent gram-positive bacterium causing community-acquired pneumonia, septic meningitis, and otitis media. The pathogenicity of *S. pneumoniae* is largely linked to its ability to produce a variety of virulence factors, among which the most relevant is pneumococcal virulence factor pneumolysin (PLY). In addition to its ability to form pores in cell membranes, PLY acts as a pathogen-associated molecular pattern by signaling via TLR4 to induce TLR4-dependent cytotoxicity in lung resident macrophages, thus further promoting the bacterial colonization of the lower respiratory tract [35, 79]. Of note, TLR4 activation acts through PI3Kγ to shift macrophages towards an anti-inflammatory scenario [24]. Mechanistically, PI3Kγ and Rab8a control cytokine production by signaling through mTOR [24], which acts as a hub downstream of TLR4 to bias cytokine responses, inhibiting NFκB-dependent transcription of pro-inflammatory cytokines, like IL-6 and IL-12, while enhancing STAT3-mediated transcription of the anti-inflammatory cytokine IL-10 [80]. Consistently, either genetic deletion or pharmacologic inhibition of PI3Kγ in mice infected with *S. pneumoniae* causes an impaired recruitment of macrophages, associated with a reduced bacterial clearance from the lungs [81]. This, in turn, results in an impaired resolution/repair process and in progressive pneumococcal pneumonia [81]. Similar results have been observed after infection by *Staphylococcus aureus*, as a higher bacterial burden is present in PI3Kγ KO mice, due to the reduced recruitment of leukocytes.

On the contrary, PI3Kγ deficiency improves the resistance against *Mycobacterium tuberculosis* in the early phase of infection, by increasing T helper IL-17+ (Th17) cells number, production of IL-17, and expression of molecules associated with Th17-cells differentiation and neutrophil recruitment [82]. These findings are in accord with previous data showing increased concentrations of IL-17 in the bronco alveolar lavage fluid of PI3Kγ KO mice challenged with intranasal instillation of lipopolysaccharide (LPS) [4].

Moreover, a deficiency in expression of PI3Kγ, along with PI3Kδ, enhances the IL-17/G-CSF axis and induces neutrophilia [83].

Of note, the crosstalk between the IL-17 signaling pathway and neutrophils recruitment seems to be time-dependent [84]: while higher neutrophil counts are protective against early tuberculosis infection [85], a pathogenic role of neutrophils during the late stages of tuberculosis has been proposed [86]. Thus, whereas pharmacological inhibition of PI3Kγ may be a suitable strategy to inhibit inflammation and limit lung damage in chronic and early-stage lung diseases, it might raise concerns in acute and late-stage infections, where it could result in an impaired host defense against high bacterial burden.

### Influenza

The role of PI3Kγ in the context of viral infections has been studied in Kaposi's sarcoma-associated herpes virus-induced tumors, where PI3Kγ is required for the viral oncogenic signaling [87]. On the other hand, PI3Kγ is also important in the regulation of innate immune responses, as well as establishment and resolution of inflammation upon influenza infection. Influenza A (IAV) and B viruses are among the most common causes of acute respiratory diseases of viral origin, accounting for three to five million cases of severe infection and up to 650,000 deaths/year worldwide [88]. In particular, the clinical manifestation of IAV infection, a highly pathogenic strain, is characterized by an excessive inflammatory response leading to lung damage [89].

Response to IAV infection can be conceptually divided in three stages, which however occur simultaneously through the course of the injury [90]. First, the immune response against the influenza virus is initiated by release of type-I and type-III interferons (IFNs), mainly produced by epithelial cells, which are primarily targeted by IAV, and by dendritic cells. Second, the innate immune system (natural killer (NK) cells, macrophages, and neutrophils), is rapidly recruited to the airways by cytokines and chemokines. Finally, specificity and memory are provided by T cells. PI3Kγ plays a crucial role in all these responses, driving production of type-I and type-III IFNs, as well as recruitment of NK and CD8+ T cells, and ultimately controlling viral titers in the infected lungs.

PI3Kγ has a pivotal role in the recruitment and survival of macrophages and neutrophils [91, 92], which, however, when excessively activated, might be harmful to the host by leading to lung damage [93, 94]. Recently, PI3Kγ has been shown to be essential after IAV infection for the control of recruitment and survival of innate immune cells and for resolution of inflammation [95]. In fact, in PI3Kγ KO mice infected by IAV, the increased production of pro-inflammatory cytokines and the accumulation of activated neutrophils in the lungs contribute to lung damage and enhanced lethality. Moreover, PI3Kγ controls leukocyte survival and resolution of inflammation, as shown by the reduced number of resolving macrophages and lower IL-10 levels in PI3Kγ KO mice infected with IAV. Keeping with that, during IAV infection, this unbalance towards pro-inflammatory signals, to the detriment of pro-resolving signals, finally results in increased lung injury in PI3Kγ KO mice.

Recently, the contribution of PI3Kγ in regulating priming of CD8+ T cells by resident dendritic cells and NK/lymphocyte migration toward chemokine stimuli in PI3Kγ KD/KD (knockdown) [96] and PI3Kγ KO mice [45], respectively, has been shown to contribute to the enhanced susceptibility to IAV infection.

Consistent with these findings, PI3Kγ orchestrates the antiviral immunity and inflammatory magnitude in response to IAV by distinct mechanisms. Therefore, targeting PI3Kγ may not be useful to treat IAV infection, possibly leading to decreased control of the infection, but might be an important diagnostic marker of disease severity. Nevertheless, the contribution of the scaffold and kinase activity of PI3Kγ have not been dissected in this context, and attention should be paid to the fact that PI3Kγ KO and KD/KD mice do not necessarily have overlapping phenotypes, as previously suggested in the heart [8, 97].

Moreover, the analysis of single-nucleotide polymorphisms (SNPs) on *PIK3CG* gene might be exploited for prognosis. Different genetic polymorphisms on genes encoding for host factors have been investigated to explain the heterogeneity of immune responses to influenza infection and disease outcomes [98]. SNPs on *PIK3CG*, or close to the gene, have been studied in genetic association studies in a number of diseases, like cardiovascular disease [99], epinephrine-induced aggregation [100] and HDL-cholesterol plasma levels [101]. Importantly, SNPs located in *PIK3CG* gene (rs17847825 and rs2230460) have been associated with disease protection in influenza A(H1N1)pdm09-infected patients [95], thus suggesting the possible use of PI3Kγ as a clinical prognostic factor.

## CONCLUSIONS

Following the initial characterization of PI3Kγ [102, 103] and the patenting of the first PI3Kγ-selective inhibitor by Novartis in 2003 for the treatment of respiratory diseases [104], drug discovery efforts in the last decade have validated the value of PI3Kγ as a promising therapeutic target, especially for inflammatory disease (for a chronological review of the patented synthetic PI3Kγ inhibitor chemotypes see [15] and [105]).

In particular, pharmacological targeting of PI3Kγ may be effective in the regulation of the immune system, and therefore in the control of airway diseases driven by an excessive inflammatory response. On the other hand, possible side effects can be expected upon long-term treatment or in the co-occurrence of infections, as highlighted by preclinical work [81]. Similarly, infections have been observed as a significant side effect of the dual PI3Kγδ inhibitor Duvelisib [106]. However, whether and how PI3Kγ selective inhibitors predispose to infections is still unknown, as only one compound selectivity targeting PI3Kγ, IPI-549 from Infinity Pharmaceuticals, has initiated clinical development so far, though as an anti-cancer agent [105]. Therefore, it cannot be excluded that inhibitors targeting PI3Kγ catalytic activity may have opposite effects in the lungs, and only clinical trials will define the nature and the extent of a therapeutic window for these drugs in pulmonary diseases, compared to the more advanced dual PI3Kγδ inhibitors.

Among these, several compounds have reached clinical trial for respiratory diseases (**Table 2**). For example, Duvelisib was a candidate for mild asthma, but further development in non-oncologic diseases has been stopped, as the primary end point (changes in maximum allergen-induced Forced Expiratory Volume 1 decrease) was missed in clinical trials. Significant effects were seen, however, on secondary end points, but at a dose potentially leading to serious adverse reactions [107]. Other dual γδ inhibitors that reached clinical development for respiratory diseases (like asthma and chronic obstructive pulmonary disease (COPD)) include RV1729 and RV6153, from RespiVert, and AZD8154, from AstraZeneca (**Table 2**). Possibly, an inhalation-based delivery of PI3Kγδ (and PI3Kγ) inhibitors could help reducing, if not overcoming, any systemic adverse effect, though impairing the response to respiratory pathogens. In line with this approach, Chiesi Farmaceutici has started a clinical study to investigate the safety, tolerability and pharmacokinetics of the inhaled CHF6523 PI3K inhibitor. As an exploratory assessment, the anti-inflammatory effect of CHF6523 on sputum and blood biomarkers in COPD subjects will be evaluated (**Table 2**).

**TABLE 2.** Clinical development of PI3Kγδ inhibitors for respiratory diseases.

**Compound**	**Developer**	**Target**	**Disease**	**Clinical Trial Identifier**	**Clinical phase**	**Status**	**Subjects enrolled**	**Duration (weeks)**	**Refs.**
IP-145 (Copiktra^®^, Duvelisib)	Verastem Oncology, licensed from Infinity Pharmaceuticals	PI3Kγδ inhibitor	Mild Asthma	NCT01653756	2	Completed	50	2	[107, 111, 112]
RV1729	RespiVert	PI3Kγδ inhibitor	Asthma Asthma COPD	NCT01813084 NCT02140320 NCT02140346	1 1 1	Completed Completed Completed	63 49 48	4 2 4	[111, 113, 114]
RV6153	RespiVert	PI3Kγδ inhibitor	Asthma	NCT02517359	1	Terminated	55	4	[105, 111]
AZD8154	AstraZeneca	PI3Kγδ inhibitor	Asthma Asthma Asthma	NCT04480879 NCT04187508 NCT03436316	1 2 1	Terminated Withdrawn Completed	10 - 78	9 - 2	[115]
CHF6523	Chiesi Farmaceutici	PI3K	inhibitor	COPD	NCT04032535	1	Recruiting	-	4	

Moreover, considering that class IB isoforms can cooperate with class IA and IB PI3Ks in controlling, downstream signaling events, dual inhibition may be desirable to achieve a relevant therapeutic effect [15]. On the other hand, from a safety perspective, a high isoform selectivity is required, especially toward PI3Kα and β, which made development of PI3Kγ inhibitors difficult, due to the high similarity between isoform sequences. Only recently, new classes of increasingly more specific inhibitors have been generated to block PI3Kγ kinase activity [108–110]. However, this approach may not discriminate between the two PI3Kγ heteromeric variants, that share the same catalytic p110γ subunit combined to different regulatory subunits, which hypothetically exert distinct biological functions [27].

Nonetheless, PI3Kγ is a multifunctional protein, which is not only involved in the modulation of the Akt/mTOR pathway through its catalytic action, but also in the inhibition of cAMP as a scaffold protein. As cAMP elevation in lungs triggers bronchodilation and anti-inflammatory responses, better definition of the protein-protein interactions driving PI3Kγ-mediated cAMP modulation might open the way to novel therapeutic options in airway diseases.

Despite efforts in developing PI3Kγ inhibitors in the last decades, only one compound, the dual γδ inhibitor Duvelisib, has received approval, and its application is limited to oncological malignancies. Therefore, a more profound understanding of the biological role of PI3Kγ variants as well as of the impact of its non-catalytic functions in signal transduction is needed in order to foster new tools, and expand fields of intervention for PI3Kγ targeting.

## Abbreviatons:

AHR: – airway hyperresponsiveness;
ASM: – airway smooth muscle;
cAMP: – cyclic adenosine monophosphate;
CF: – cystic fibrosis;
CFTR: – CF transmembrane conductance regulator;
GPCR: – G-protein coupled receptor;
COPD: – chronic obstructive pulmonary disease;
IAV: – influenza A;
IFN: – interferon;
IL: – interleukin;
IPF: – idiopathic pulmonary fibrosis;
KD: – knockdown;
KO: – knockout;
LPS: – lipopolysaccharide;
MIF: – migration inhibitory factor;
NK: – natural killer;
PI3K: – phosphatidyl inositol 3 kinase;
PLY: – pneumolysin;
SNP: – single-nucleotide polymorphisms;
TLR: – Toll-like receptor;
VILI: – ventilation-induced lung injury.
